# Connexin 43 is an independent predictor of patient outcome in breast cancer patients

**DOI:** 10.1007/s10549-018-5063-9

**Published:** 2018-11-24

**Authors:** Maria Chasampalioti, Andrew R. Green, Ian O. Ellis, Emad A. Rakha, Andrew M. Jackson, Ian Spendlove, Judith M. Ramage

**Affiliations:** 1 Cancer Immunology Group, Division of Cancer and Stem Cells, School of Medicine, University of Nottingham, Nottingham, UK; 2 Division of Cancer and Stem Cells, Nottingham Breast Cancer Research Centre, School of Medicine, University of Nottingham, Nottingham, UK; 3 Department of Pathology, Nottingham University Hospitals NHS Trust, Nottingham City Hospital, Nottingham, UK; 4 Host-tumour interactions Group, Division of Cancer and Stem cells, School of Medicine, University of Nottingham, Nottingham, UK; 5 Academic Unit of Clinical Oncology, University of Nottingham, Nottingham City Hospital, Nottingham, NG5 1PB UK

**Keywords:** Connexin 43, Tissue microarray, Tumor biomarker

## Abstract

**Purpose:**

Gap junctions are specialized membrane structures that form channels between adjacent cells allowing cell communication. Gap junctions and specifically Connexin 43 (Cx43) are down-regulated in cancer; however, there are contrasting reports on how this effects breast cancer patient survival. This paper is the first large-scale tissue microarray analysis of Cx43 expression in breast cancer patients with an associated clinical long-term follow-up.

**Methods:**

Using a validated TMA of 1118 primary breast cancers, coupled to a comprehensive database of clinicopathological variables, the expression levels and subcellular localisation of Cx43 was assessed by immunohistochemistry. Its impact in terms of survival, distant metastasis-free survival, and clinicopathological variables was determined.

**Results:**

Patients whose tumors expressed high levels of Cx43 had significantly better survival (*p* < 0.001) than patients with low levels. High Cx43 expression within tumors was associated with an 18-month survival advantage. Loss of Cx43 expression was associated with markers of poor prognosis, namely large tumor size, high grade, high proliferation status, high pleomorphism, high mitosis, poor Nottingham Prognostic Index (NPI), and triple negative tumors. Cx43 expression was independent of tumor size, grade, stage and ER-status in predicting poor survival on multivariate analysis (*p* = 0.004).

**Conclusion:**

Connexin 43 (Cx43) is an independent predictor of breast cancer survival and distant metastasis-free survival. High expression of Cx43 was seen in only 13% of tumors, suggesting that drugs to increase Cx43 expression may result in prolonged patients survival.

**Electronic supplementary material:**

The online version of this article (10.1007/s10549-018-5063-9) contains supplementary material, which is available to authorized users.

## Introduction

Connexin 43 (Cx43) has dual functions due to its involvement in cell-to-cell communication through gap junctions and in maintenance of homeostasis. Gap junctions are specialized membrane structures that permit the formation of channels between adjacent cells. Two hemi-channels (made up of 6 connexins) pair to form an intracellular gap junction channel allowing the passage of second messengers, ions and other small molecules (< 1000 Da), in a process known as gap junctional intracellular communication (GJIC) [1]. The carboxyl-terminus (C-terminus) regulatory domain of Cx43 is located in the cytoplasm. This is the primary site of its protein:protein interactions and phosphorylation and has been demonstrated to have a role in its intracellular functions such as proliferation, apoptosis, and migration [2].

There have been reports that expression levels of Cx43 are altered in breast cancer, however, there is controversy over its role in patient survival [3]. In a small study of 32 patient samples, Cx43 was shown to correlate with poor survival in breast cancer [4]. In contrast, a study of invasive breast carcinoma by Conklin et al. showed no correlation of Cx43 expression with patient outcome [5]. More recently a meta-analysis of transcriptome data has shown an association between loss of Cx43 expression and poor prognosis [6]. But again, in apparent contrast, Stolevoc et al. found that RNA expression was associated with increased patient death and recurrence [7] in primary breast cancer.

High levels of expression of Cx43 on metastatic cells in the lymph node [8] and brain [7] have led to the suggestion that Cx43 plays a role in metastasis. There is controversy here as well, as in apparent contrast, where Cx43 expression is down-regulated [9–11], in animal models an increase in metastasis is associated with a loss of Cx43. This is reinforced by experiments in Cx43 knockout mice [12].

As Cx43 has an underlying importance in controlling a range of cellular functions, it is perhaps not surprising that it is dysregulated in cancer. Cx43 is expressed in normal breast tissue [13], where it localized to the plasma membrane [14] however, in tumors there is a loss or mislocalisation of Cx43 from the plasma membrane to cytoplasm [8]. This has led to the debate of whether the tumor suppressor function of Cx43 is gap junction dependent or independent [9, 11]. There is therefore a requirement to further clarify the relevance of Cx43 localisation to survival.

We hypothesized that downregulation of Cx43 in tumors would lead to increased metastasis and poor survival in breast cancer patients. This paper is a large-scale tissue microarray analysis of Cx43 expression in breast cancer patients with an associated long-term follow-up. The expression of Cx43 in both the membrane and the cytoplasm were investigated due to previous reports of the importance of location. We demonstrate that Cx43 expression in either the membrane or cytoplasm is not only an independent predictor of survival, it is also an independent predictor of distant metastasis-free survival in breast cancer.

## Materials and methods

Ethical approval was granted by the Nottingham Research Ethics Committee (Nottingham Hospital Trust numbers Q1020402 and GS020402, REC202313). The samples used in this study have been described previous publications [15]. In an attempt to overcome some of the reporting deficiencies inherent in prognostic tumor marker studies, we followed the Reporting recommendations for tumor MARKer prognostic studies (REMARK) [16].

### Study patients

This retrospective study was based on a series of 1118 patients with primary invasive breast carcinomas who were diagnosed from 1987 to 1998 and entered onto the Nottingham Tenovus Primary Breast Carcinoma series. All pathological characteristics were recorded at time of presentation including histologic tumor grade, histologic tumor type, tumor size, Nottingham prognostic index (NPI), vascular invasion, and protein expression. All patients had standard surgical treatment of mastectomy or local excision with radiotherapy. Breast cancer-specific survival and distant-free metastasis was measured in years from the date of the primary surgical treatment to the time of death of breast cancer.

### Tissue microarrays and immunohistochemistry

At the time of resection, all tumors were managed in a standardized fashion, with immediate incision and fixation in neutral-buffered formalin to minimize any diffusing problems, followed by processing through to embedding in paraffin wax. Breast cancer tissue microarrays were prepared as previously described [15]. In brief, tissue cores with a diameter of 0.6 mm were punched from the representative tumor regions of each donor block. Cores were precisely arrayed into new recipient paraffin block using a tissue microarrayer (Beecher Instruments). Immunohistochemistry of 0.4 µm thick sections was performed utilizing the Novolink polymer detection system (Leica Biosystems, UK). Briefly tissue slides were deparaffinised with xylene and rehydrated through three changes of alcohol. Citrate buffer was used for antigen retrieval (20 min in microwave). To all slides 100 µl of Peroxidase Block (Novolink Kit) was added for 5 min, followed by two TBS washes and 100 µl of protein block for 5 min. After a further two TBS washes 100 µl of anti-Cx43 antibody (Invitrogen™ Thermo-Fischer Scientific previously optimized at 12.5 µg/ml) was applied and slides were incubated for 1 h. Slides were washed twice with TBS and then incubated for 30 min with 100 µl Rabbit anti mouse IgG (Novolink kit). Then washed twice with TBS and incubated for 30 min with 100 µl of Novolink Polymer of anti-rabbit Poly-HRP-IgG. Finally the sections were washed twice with TBS and 100 µl of DAB working solution (1:20 DAB chromogen in DAB substrate buffer (Novolink Kit)) was added for 5 min. After washing the slides twice with TBS they were counterstained with 100 µl of Novolink haematoxylin for 6 min dehydrated and a coverslip applied. Negative (omission of the primary antibody) and positive controls (anti-β-2 microglobulin) were included.

### Evaluation of immunohistochemistry staining

Assessment of staining was based on semi quantitative approach using light microscopy. Cytoplasmic and membranous expression of Cx43 was scored separately in each core. Membrane expression was scored as follows in terms of intensity as 0, 1, 2, or 3 for negative, weak, moderate and strong respectively. For the cytoplasmic scoring where percentage of tumor staining could readily be determined H-score were used to determine the cytoplasmic staining with both the intensity and percentage of staining assessed. Staining intensity was scored as 0, 1, 2, or 3 for negative, weak, moderate and strong respectively and multiplied by percentage staining (range 0–300).

### Statistical analysis

The X-tile bioinformatics software version 3.4.7 was used for determination of the optimal cut-off points for Cx43 expression based on prediction of breast cancer-specific survival. The Statistical analysis was performed using the SPSS 21.0 statistical software (SPSS, USA). Univariate and multivariate analyses were performed by chi-squared, log rank, and Cox regression analysis, respectively. Survival curves were analyzed by Kaplan–Meier curves. A *p*-value < 0.01 was considered significant.

## Results

### Low expression of Cx43 in primary breast cancer

**Fig. 1:**
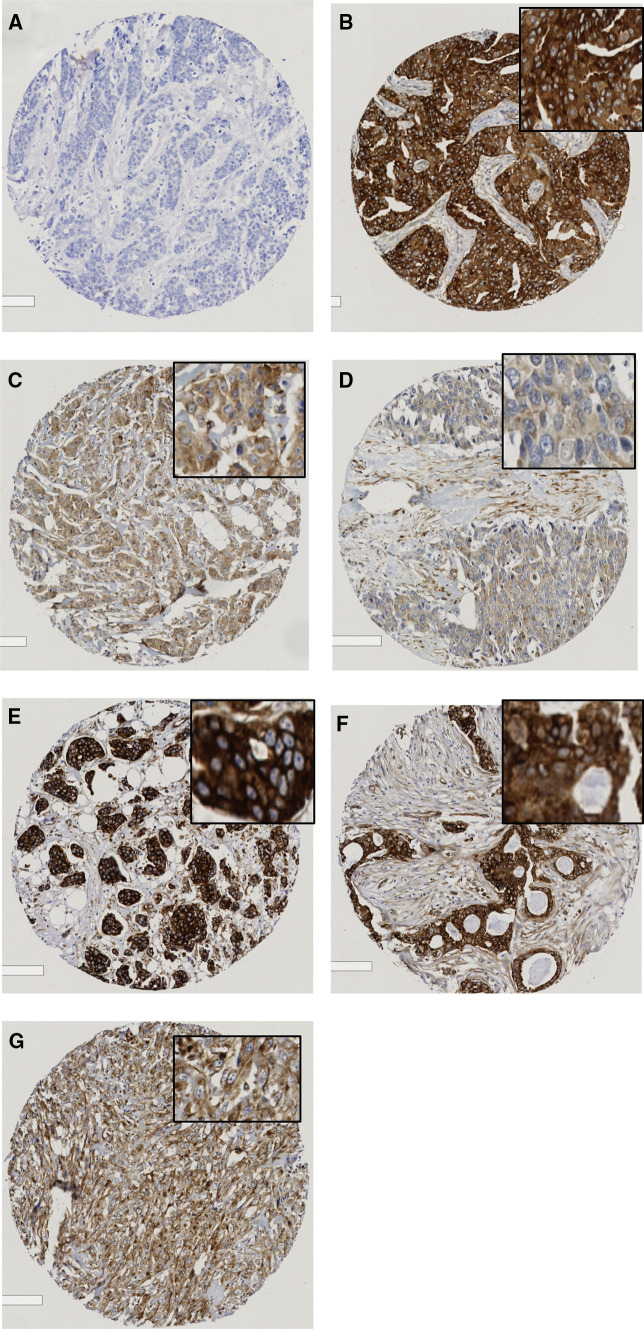
Photomicrographs of breast TMA scores immunohistochemically stained for Cx43 (**a**–**g**). The level of Cx43 expression ranged from **b** high, **c** moderate, **d** weak to (**a**) negative for cytoplasm and from **e** high, **f** moderate, **g** weak for membrane

A tissue microarray (TMA) series of 1980 breast patients was immunoprobed for Cx43 expression. From the original series 1118 (48%) had sufficient cores for scoring to be performed, the remaining cores were lost during antigen retrieval or did not have sufficient tumor cells present. Supplementary table 1 demonstrates that the clinicopathological variables of the cohort did not vary greatly from the original whole dataset. Intensity of staining was categorized as high, moderate, weak (Fig. 1b–d), or negative (Fig. 1a) in the cytoplasm and the membrane (Fig. 1e–g).

For cytoplasmic expression where the percentage staining of the tumor could be readily determined, the intensity (Fig. 1 c–e) was multiplied by percentage staining to give H-scores. Cytoplasmic Cx43 expression was dichotomised as low (H-score range 0–180) and high (H-score range 181–300). For membranous Cx43 expression, where percentage staining was not readily determined, the high, moderate, weak, and negative cores were further divided into 2 categories: low [combining negative and weak (Fig. 1a, g)] and high [combining moderate and high (Fig. 1e, f)] expression. The majority of tumors had low Cx43 membranous expression [87% cases; mean score of 0.92 (range 0–3)] and low expression (73% of cores) in the cytoplasm (mean H-score of 106).

### Low Cx43 expression associated with known clinicopathological markers of poor prognosis

Low levels of Cx43 expression in either the membrane or the cytoplasm were significantly associated with known markers of poor prognosis: tumor size (membrane *p* = 0.02; cytoplasm *p* = 0.001), higher grade (*p* < 0.0001), high nuclear and cellular pleomorphism (membrane *p* = 0.002; cytoplasm *p* < 0.0001), high mitosis frequency (*p* < 0.0001), and poor NPI (*p* < 0.0001) (Table 1). In terms of tumor biology, there was an association between low Cx43 and ER, PgR, and HER2 negativity and triple negative tumors (all *p* < 0.0001). Low levels of expression were associated with high proliferation (ki67 + *p* < 0.0001) levels. Low levels of membrane expression were also significantly associated with pre-menopausal and younger patients (Table 1).

**Table 1:** Association between the expression of cytoplasmic and membrane Connexin43 (Cx43) and Clinicopathologic features in primary breast cancer

Clinicopathological variables	Cytoplasmic expression			Membrane Expression		
	Low (%)	High (%)	*p*-value	Low (%)	High (%)	*p*-value
Menopause						
Pre	326 (75)	112 (25)		371 (84)	67 (15)	
Pos	491 (73)	177 (26)	0.732	595 (89)	73 (10)	0.033
Age diagnosis						
≤ 50	295 (74)	103 (26)		336 (84)	62 (16)	
> 50	525 (73)	192 (27)	0.744	635 (89)	82 (11)	0.048
Tumor size						
≤ 2.0 cm	368 (69)	166 (31)		452 (85)	82 (15)	
> 2.0 cm	452 (78)	129 (22)	0.001	519 (89)	62 (11)	0.02
Grade						
1	97 (55)	80 (45)		135 (76)	42 (24)	
2	246 (65)	130 (35)		316 (84)	60 (16)	
3	477 (85)	85 (15)	< **0.0001**	519 (92)	145 (13)	< **0.0001**
Tubule formation						
1 (majority of tumor,< 75%)	28 (48)	30 (52)		37 (64)	21 (62)	
2 (moderate degree, 10–75%)	241 (65)	131 (35)		304 (82)	68 (18)	
3 (little or none, < 1% none, < 10%)	523 (80)	290 (27)	< **0.0001**	599 (92)	53 (8)	< **0.001**
Pleomorphism						
1 (small, regular uniform cells)	15 (68)	7 (32)		16 (73)	6 (27)	
2 (moderate increase in size and variablility)	253 (62)	156 (38)		341 (83)	68 (17)	
3 (marked variation)	524 (80)	127 (20)	< **0.0001**	583 (90)	68 (10)	0.002
Mitosis						
1	208 (60)	141 (40)		284 (81)	65 (15)	
2	151 (67)	75 (33)		185 (82)	41 (18)	
3	433 (85)	74 (15)	< **0.0001**	940 (87)	142 (13)	< **0.0001**
Tumor type						
Ductal	690 (73)	251 (27)		818 (87)	123 (13)	
Lobular	71 (75)	24 (25)		88 (93)	7 (7)	
Medullary-like	22 (96)	1 (4)		23 (100)	0 (0)	
Miscellaneous	4 (80)	1 (20)		4 (80)	1 (20)	
Special type	33 (65)	18 (35)	0.091	38 (74)	13 (25)	0.009
Vascular invasion						
Absent	521 (72)	201 (28)		626 (87)	96 (13)	
Present	295 (76)	93 (24)	0.164	341 (88)	47 (12)	0.575
Ki67						
Low	180 (63)	105 (37)		241 (85)	44 (15)	
High	510 (79)	138 (21)	< 0.0001	578 (89)	70 (11)	
Nodal stage						
1	469 (71)	190 (29)		570 (86)	89 (13)	
2	275 (75)	91 (25)		318 (87)	48 (13)	
3	76 (84)	14 (15)	0.019	82 (91)	8 (9)	0.473
NPI						
Good	184 (57)	140 (43)		261 (81)	63 (19)	
Moderate	473 (79)	124 (21)		532 (89)	65 (11)	
Poor	163 (84)	31 (16)	< **0.0001**	178 (92)	16 (8)	< **0.0001**
ER-status						
Negative	273 (96)	12 (4)		282 (99)	3 (1)	
Positive	536 (65)	283 (35)	< **0.0001**	680 (83)	139 (17)	< **0.0001**
PgR-status						
Negative	407 (91)	39 (9)		429 (96)	17 (4)	
Positive	380 (61)	246 (39)	< **0.0001**	506 (81)	120 (19)	< **0.0001**
HER2 status						
Negative	654 (70)	276 (30)		796 (86)	134 (14)	
Positive	126 (93)	9 (7)	< **0.0001**	132 (98)	3 (2)	< **0.0001**
Triple negative						
Non-triple	600 (68)	284 (32)		745 (84)	139 (16)	
Triple negative	188 (94)	11 (5)	< **0.0001**	196 (98)	3 (1)	< **0.0001**
Basal phenotype						
Negative	569 (70)	241 (30)		690 (85)	120 (15)	
Positive	235 (84)	46 (16)	< **0.0001**	262 (93)	19 (7)	< **0.0001**

Bold indicates significant *p*-value (< 0.05). Clinicopathological variables

*NPI* Nottingham prognostic index

### Low Cx43 expression is associated with poor patient outcome

**Fig. 2:**
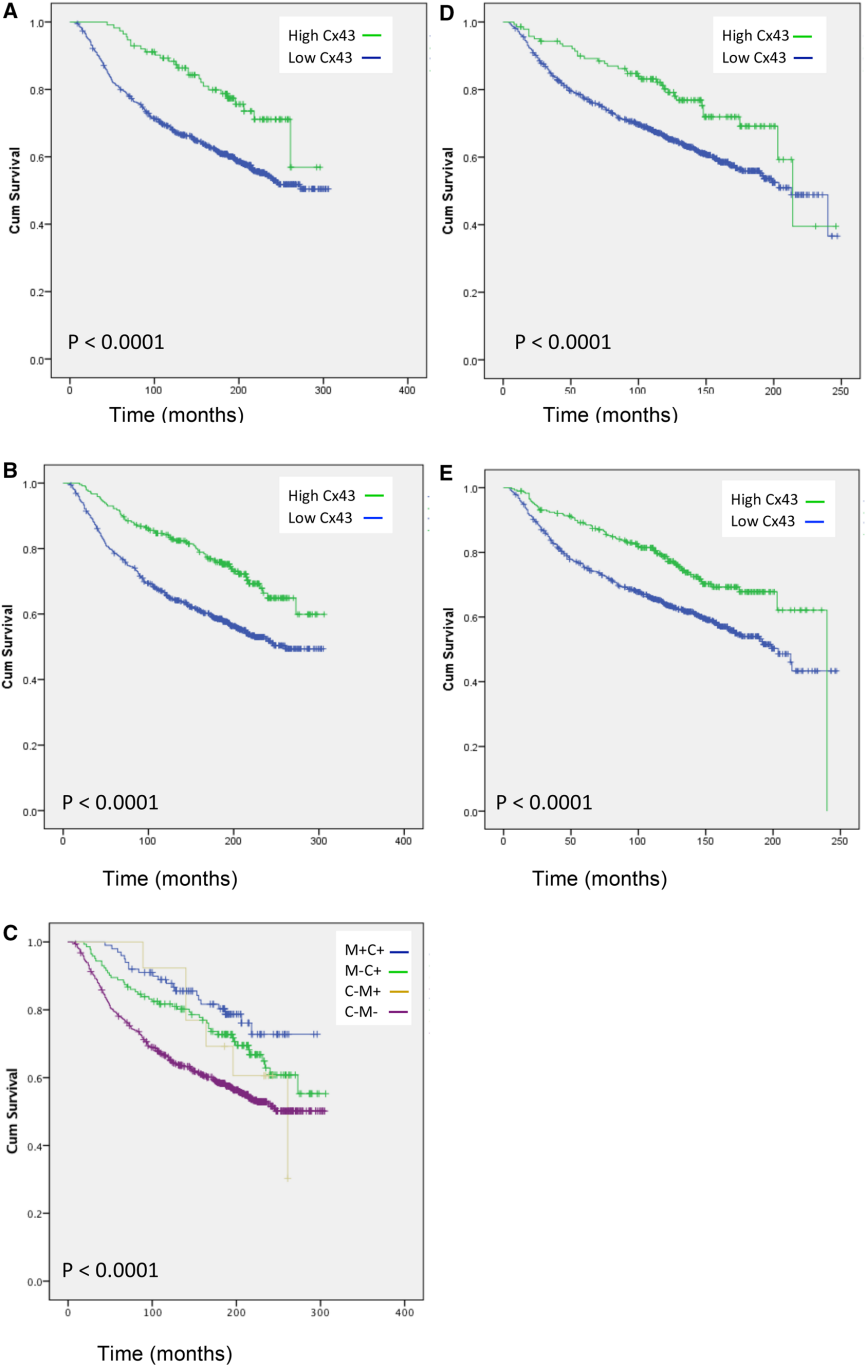
Low Cx43 expression associated with poor breast cancer patient survival. Kaplan–Meier curves of membrane CX43 expression association with survival (**a**) cytoplasm Cx43 staining association with survival (**b**), Cx43 membrane (M) and Cx43 cytoplasmic (C) co-expression association with survival (**c**) membrane Cx43 expression and distant metastasis-free survival (**d**) and Cytoplasmic Cx43 expression and distant metastasis-free survival (**e**)

Low expression of both membranous and cytoplasmic Cx43 showed a statistically significant association with breast cancer-specific survival (*p* < 0.0001; Fig. 2a, b; Table 2) with survival at 206 months compared to 244 months for low membrane expression and 200 months compared to 243 months for low cytoplasmic expression. However, patients whose tumors had high Cx43 expression in both the cytoplasm and the membrane had a better survival outcome than those that were only positive in the cytoplasm (252 months compared to 235 months, *p* < 0.0001; Fig. 2c). No expression of Cx43 was the worst prognosis (200 months). There were limited numbers of only high Cx43 membrane expression.

**Table 2:** Mean survival time and mean distant metastasis-free survival in relation to Cx43 expression in the membrane or the cytoplasm

Expression	Cx43 membrane expression				Cx43 cytoplasmic expression			
	Estimate (months)	95% confidence interval		*p* value	Estimate (months)	95% confidence interval		*p* value
		Lower bound	Upper bound			Lower bound	Upper bound	
Mean survival time								
High	244	227	262	< 0.0001	243	231	256	< 0.0001
Low	206	198	214		200	192	209	
Overall	212	204	219		212	204	219	
Distant metastasis-free survival (mean)								
High	188	171	205	< 0.0001	186	178	199	< 0.0001
Low	166	159	173		162	154	169	
Overall	169	163	175		169	163	175	

### Low Cx43 expression associated with shortened distant metastasis-free survival

Moreover, low Cx43 membranous expression predicted shortened distant metastasis-free survival giving a 14 month disadvantage than high expression (166 months compared with 188 months). Similarly, low Cx43 cytoplasmic expression gave 15-month survival disadvantage over high expression (162 months compared to 186 months; *p* < 0.0001) (Fig. 2d, e).

### Cx43 expression is an independent predictor of patient survival and distant metastasis-free survival

Multivariate Cox regression analysis demonstrated that Cx43 expression, either membranous or cytoplasmic, was independent of tumor size, grade, stage, and ER-status in predicting breast cancer-specific (membrane *p* = 0.003; cytoplasmic *p* = 0.004) and metastasis-free survival (membrane *p* = 0.003; cytoplasmic *p* = 0.005) (Table 3).

**Table 3:** Multivariate analysis of Cx43 expression compared with tumor stage, grade, size, and ER-status for breast cancer specifc survival and distant metastasis free survival

Breast cancer specific survival								
Expression	Membrane expression				Cytoplasmic expression			
	Hazard ratio	95% confidence interval			Hazard ratio	95% confidence interval		
		Lower bound	Upper bound	*P* value		Lower bound	Upper bound	*P* value
Tumor stage	1.866	1.605	2.169	0.0001	1.854	1.595	2.155	0.0001
Tumor grade	1.639	1.367	1.964	0.0001	1.597	1.331	1.918	0.0001
Tumor size	1.339	1.076	1.666	0.009	1.330	1.069	1.654	0.011
ER-status	1.214	0.949	1.554	0.122	1.242	0.968	1.592	0.088
Cx43 expression	0.546	0.546	0.364	**0.003**	0.0664	0.664	0.502	**0.004**

Distant metastasis free survival								
Expression	Membrane expression				Cytoplasmic expression			
	Hazard ratio	95% confidence interval			Hazard ratio	95% confidence interval		
		Lower bound	Upper bound	*P* value		Lower bound	Upper bound	*P* value
Tumor stage	1.943	1.682	2.253	0.0001	1.927	1.668	2.234	0.0001
Tumor grade	1.508	1.270	1.777	0.0001	1.469	1.235	1.734	0.0001
Tumor size	1.345	1.092	1.669	0.009	1.341	1.089	1.664	0.007
ER-status	1.231	0.958	1.570	0.122	1.260	1.254	0.978	0.069
Cx43 expression	0.638	0.449	0.909	**0.003**	0.684	0.528	0.528	**0.005**

Bold indicates *P* value for Cx43 expression as an independent predictor

## Discussion

Previous studies have highlighted that Cx43 expression is dysregulated in breast cancer, but there is no consensus on its role in patient survival and prediction of metastasis. In this study of over 1000 breast cancer patients with 30 years follow-up, low Cx43 expression was associated with poor patient prognosis and outcome. Indeed, low expression correlated with a wide range of established clinicopathological markers of poor prognosis such as larger tumor size, higher grade, poor NPI, and triple negative status. Therefore, the more aggressive tumors had low/ no expression of Cx43. Low expression of Cx43 was an independent predictor of patient survival. Importantly, to the best of our knowledge, this is also the first report of Cx43 as an independent predictor of distant metastasis-free survival.

In contrast to previous studies that used significantly lower numbers of patient samples, this study analyzed over 1000 primary tissue samples and we were therefore able to firmly establish that Cx43 is an independent predictor of survival. This is in agreement with a recent meta-analysis study of RNA levels in breast cancer, which also demonstrated, that Cx43 was an independent predictor of poor survival [6]. However, these results are in contrast to a paper by Conklin et al. using invasive breast carcinoma [17], which showed no correlation of Cx43 protein expression with patient outcome. This paper primarily observed Cx43 expression in the cytoplasm and did not observe membrane expression. In our analysis expression of Cx43 in both membrane and cytoplasm was associated with the best patient survival. It may have been that the Conkilin et al. analysis had a larger portion of higher grade patients in their samples but as there are no clinical characteristics given in their paper we are unable to compare.

Cx43 is hormone responsive [18, 19], and this may explain the positive correlation of Cx43 expression with both ER- and PgR-status. These findings are in agreement with Conkil et al. who showed that Cx43 correlated with ER-status [17]. Cx43 expression was inversely correlated with Ki67, consistent with other data [17] and this would suggest that loss of Cx43 resulted in increased proliferation of cells.

There has been debate over the expression of Cx43 at different stages of cancer [20]. This study clarifies the correlation of aggressive tumors/triple negative tumors with low Cx43 expression. Moreover, our analysis is the first to report that Cx43 is an independent predictor of metastasis-free survival and thereby supports the hypothesis of Mao et al. that gap junctions serve as an “intracellular glue” to suppress metastasis [21]. An elegant animal model study by Saunders et al. showed that transfection of the metastatic suppressor gene (BRMS1) into a human breast cancer metastatic cell line restored intracellular gap junctional communication and correlated with improved survival due to less metastasis [22]; thereby, highlighting the requirement for Cx43 to prevent metastasis. This is also consistent with mouse models where a high metastatic potential correlated with loss of IJC [9–11] and also in Cx43 knockout mice [12] which had higher metastasis in tumor models than wild-type mice from transplantable tumors. Therefore, indicating that higher expression of Cx43 is important in preventing metastasis.

There is some apparent controversy in the literature regarding the role of Cx43 in metastasis due to the reports of high Cx43 in metastatic tissue [8] of cancer patients. However, the literature would be consistent with downregulation of Cx43 being required for tumors to metastasise and that once they have metastasised they upregulate Cx43 as postulated by Stoletov et al. [7]. Indeed Elzarrad et al. [23] state that some stages of tumorigenesis and metastasis (uncontrolled cell division and cellular detachment) require loss of gap junctions, while other stages (intravasation, endothelial attachment, and vascularization) require increased cell–cell contact. They hypothesize that this is a multi-stage scheme where Cx43 is involved centrally as a cell adhesion molecule mediating metastatic tumor attachment to pulmonary endothelium. Furthermore, the Knockdown of Cx43 has also been associated with activation of the Wnt/β-catenin signaling pathway, which is known to have a central role in cancer biology [24]. Indeed, blocking of the Wnt/β signaling suppresses breast cancer metastasis. It is tempting to speculate that downregulation of Cx43 results in increased Wnt/β signaling leading to increase metastasis.

The membrane localization of Cx43 expression has been associated with gap junctional communication between cells, whereas the cytoplasmic localization has been associated with cell homoeostasis (such as controlling cell proliferation and apoptosis). Our study analyzed the expression of both membranous and cytoplasmic Cx43. The majority of tumors had low Cx43 expression (87%) in the membrane suggesting that the majority of tumors had lost expression from the membrane. Previous reports have shown a mislocalisation of expression of Cx43 from the plasma membrane to the cytoplasm in breast cancer [8]. We demonstrated that although Cx43 was detected in the cytoplasm this was at low levels in the majority of the tumors (73%). It has been hypothesized that cytoplasmic localization of Cx43 points to decreased GJIC. In human breast carcinomas tumor cell lines [3] the localization of Cx43 to the cytoplasm was not associated with gap junctional communication [20]. As Cx43 is not normally expressed in the cytoplasm our work is consistent with the hypothesis that Cx43 is mislocalised to the cytoplasm in breast cancer. In this study high expression of Cx43 in both the membrane and the cytoplasm, gave better survival that membrane only, which was better than no expression. Membrane only gave the worst prognosis, however we believe this was due to insufficient samples in this category. Therefore, this may suggest that as the tumor progresses it first dysregulates Cx43 to the cytoplasm and then loses Cx43 expression in the membrane before losing it completely.

In conclusion, Cx43 expression in breast cancer is a prognostic marker of survival and distant metastasis-free survival. Low levels of Cx43 give shorter patient survival; therefore, therapies to enhance its expression could lead to improvement in breast cancer patients survival. This has been demonstrated in animal models where retroviral delivery of Cx43 to tumors has been shown to increase survival [25]. Moreover, the drugs Geinstein and quercetin have been shown to increase Cx43 and suppress the growth of human breast cancer cell lines [17]. It would warrant future work to investigate Cx43 as a drugable target in breast cancer.

## Electronic supplementary material

Below is the link to the electronic supplementary material.

Supplementary material 1 (PDF 79 KB)
