# Supplementary material for: Connexin 43 is an independent predictor of patient outcome in breast cancer patients
Source: Breast Cancer Res Treat. 2018 Nov 24;174(1):93–102. doi: 10.1007/s10549-018-5063-9 (PMC6418069; doi:10.1007/s10549-018-5063-9)
Supplement: Supplementary file 1 — Supplementary material 1 (PDF 79 KB) [file 10549_2018_5063_MOESM1_ESM.pdf]

Supplementary table 1: Clinicopathological variables of whole database and Cx43 analysed samples A TMA series of 1,980 breast cancer patients was immunoprobed for Cx43 expression. From the original series 1118 (56%) had sufficient cores for scoring to be performed.

Clinicopathological variables	Whole database	Cx43 analysed samples
**Age diagnosis**		
<=50	667 (34.4%)	398 (35.6%)
>50	1274 (65.6%)	717 (64.1%)
**Tumor size**		
<=2.0 cm	1010 (52%)	534 (48%)
> 2.0 cm	931 (48%)	581 (52%)
**Grade**		
1	440 (19.9%)	177 (15.8%)
2	757 (34.2%)	376 (33.6%)
3	1017 (45.9%)	562 (50.3%)
**Tumor type**		
Ductal	1559 (80.3%)	941 (84.2%)
Lobular	215 (11.1%)	95 (8.5%)
Medullary-like	44 (2.3%)	23 (2.1%)
Miscellaneous	10 (0.5%)	5 (0.4%)
Special type	113 (5.8%)	51 (4.6%)
**Vascular invasion**		
Absent	1326 (68.8%)	722 (64.6%)
Present	602 (45.4%)	388 (34.7%)
**Nodal stage**		
1	1501 (66%)	659 (58.9%)
2	595 (26.2%)	366 (32.7%)
3	177 (7.8%)	90 (8.1%)
**NPI**		
Good	645 (33.2%)	324 (29%)
Moderate	997 (51.4%)	597 (53.6%)
Poor	299 (15.4%)	194 (17.4%)
**ER-status**		

Negative	547 (25.6%)	285 (25.8%)
Positive	1592 (64.1%)	819 (74.2%)
**PgR-status**		
Negative	762 (40.7%)	446 (41.6%)
Positive	1111 (59.3%)	626 (58.4%)
**HER2 status**		
Negative	1648 (87.8%)	930 (87.3%)
Positive	230 (12.2%)	135 (12.7%)
**Triple negative**		
Non-triple	1710 (84.2%)	884 (81.6%)
Triple negative	322 (15.8%)	199 (18.4%)
**Basal phenotype**		
Negative	1405 (74.7%)	810 (74.2%)
Positive	475 (25.3%)	281 (25.8%)
